# Thrombin Generation and Platelet Function in ICU Patients Undergoing CVVHD Using Regional Citrate Anticoagulation

**DOI:** 10.3389/fmed.2021.680540

**Published:** 2021-06-14

**Authors:** Marion Wiegele, Lukas Infanger, Conrad Lacom, Stefan Koch, Andreas Baierl, Eva Schaden

**Affiliations:** ^1^Department of Anaesthesia, Critical Care and Pain Medicine, Division of General Anaesthesia and Intensive Care Medicine, Medical University of Vienna, Vienna, Austria; ^2^Department of Statistic and Operations Research, University of Vienna, Vienna, Austria

**Keywords:** acute kidney injury, renal replacement therapy, hemostasis, multiple electrode aggregometry, thrombin generation assay

## Abstract

**Background:** To investigate pro- and anticoagulant alterations in uremic critically ill patients prior to and during continuous renal replacement therapy. In addition to the conventional thrombin generation assay (TGA), we performed a thrombomodulin-modified variant to better elucidate procoagulant imbalances. Platelet function was determined via multiple electrode aggregometry (MEA) to round off hemostatic analysis.

**Methods:** We prospectively enrolled patients at surgical intensive care units (ICU) with acute kidney injury undergoing continuous veno-venous hemodialysis using regional citrate anticoagulation. TGA and platelet function testing were performed at baseline (≤ 12 h prior to continuous renal replacement therapy) and on 3 consecutive days (day A–C) of extracorporeal therapy.

**Results:** We did not observe significant changes in thrombin generation after start or during renal replacement therapy. Ratios of endogenous thrombin potential in patients were significantly increased (*p* < 0.001) compared to standardized plasma of healthy donors confirming the assumed procoagulant alterations in ICU patients. Test results of the conventional TGA differed significantly (*p* < 0.05) from those of the thrombomodulin-modified assay. The area under the curve remained below MEA reference values during the entire observation period, indicating a persistent reduction in platelet function.

**Conclusion:** In summary, in-depth analysis using standard and modified TGA, as well as calculation of endogenous thrombin potential (ETP) ratios, revealed no further aggravation of the procoagulatory shift in the critically ill patient during CVVHD using regional citrate anticoagulation. MEA ruled out the potential impact of platelets.

**Clinical Trial Registration:** German Clinical Trials Register (DRKS00004336), 29 August 2012; www.drks.de.

## Introduction

Critically ill patients are at high risk of venous thromboembolism (VTE) (1–3). At the same time, comorbidities and therapeutic interventions often contribute to an increased risk of bleeding (4–8).

Conventional coagulation assays do not sufficiently depict hemostatic imbalances, whether hypo- or hypercoagulable (6, 9). Hence, over the past decade huge efforts have been made to find alternative tests which offer more detailed insight into the hemostasis of patients admitted to the intensive care unit (ICU).

Viscoelastic tests such as rotational thrombelastometry or thrombelastography, were the first tests better able to elucidate hypo- and hypercoagulability in critically ill patients (10). Recently, there has been more interest in thrombin generation assays (TGA) for this special cohort. TGA evaluate the individual's potential to generate thrombin, which triggers further procoagulant processes (11). When recombinant thrombomodulin (TM) is added during the procedure, TGA can also be used to investigate the contribution of endogenous anticoagulants to hemostasis (12). This has not yet been described in critically ill patients. Multiple electrode aggregometry (MEA) to investigate platelet function rounds off the options for detailed hemostatic analysis (13, 14).

Multifaceted hemostatic imbalances are an issue especially in critically ill patients requiring renal replacement therapy (CRRT): the contact of blood with foreign surfaces activates procoagulant processes (9) further aggravating the *per se* prothrombotic risk related to critical illness (1–3), whereas uremic platelet dysfunction is accompanied by a bleeding tendency (9, 15, 16). Caught between an increased risk of bleeding complications on the one hand and thrombosis on the other hand, anticoagulation of the extracorporeal circulation is a particular challenge. Citrate allows regional anticoagulation of the extracorporeal circuit without increasing the bleeding risk (9).

The chronological interplay of pro- and anticoagulant forces during treatment of uremia is not yet fully understood. Thus, the aim of the study was to investigate hemostatic alterations beyond conventional coagulation tests. In addition to conventional TGA, we performed a TM-modified TGA to better elucidate anticoagulant forces. Results of MEA completed the overall hemostatic picture.

## Materials and Methods

This prospective observational trial was performed in accordance with the principles of the 1964 Declaration of Helsinki and its later amendments. Approval was granted by the Ethics Committee of the Medical University of Vienna (4 July 2012, No. 1416/2012). Informed consent was obtained from all participants included in the study. All patients consented to the publication of anonymized data. This trial was registered at the German Clinical Trials Register (no. DRKS00004336) prior to the start of patient recruitment (August, 29 2012).

### Patients and Sample Collection

We screened adult patients (>18 years) with acute kidney injury indicating CRRT for eligibility at three surgical ICUs at the Medical University of Vienna, Austria, between February 7, 2013 and November 21, 2018. Patients with impaired hemostasis (due to e.g., known coagulation disorders, therapeutic anticoagulation, major bleeding, severe liver dysfunction) prior to or at the start of CRRT were excluded. Continuous veno-venous hemodialysis (CVVHD) with regional citrate anticoagulation was performed via multiFiltrate® (Fresenius Medical Care AG) using commercially available equipment and solutions (Ultraflux AV 1000s, Ci-Ca dialysat K2, sodium citrate 4%, 0.5 M CaCl_2_) (17). Therapy was started with default settings defined for adults (blood flow-effluent flow ratio: 1:20; calcium: 1.7 mmol L^−1^; citrate: 4 mmol L^−1^) (17). We adjusted flow rates when indicated by metabolic disturbances following a standardized, previously published protocol (17). Furthermore, we determined total calcium levels once daily, to screen for citrate accumulation. All patients received VTE prophylaxis (enoxaparin 40 mg once daily) in line with current guidelines recommending LMWH chemoprophylaxis (3). Coagulation assays were performed at baseline (≤ 12 h prior to CVVHD) and on 3 consecutive days (day A–C; accounting for the average life-span of the hemofilter) at trough levels of LMWH treatment. Demografic data including the Caprini Score (18) for VTE risk assessment, medical history, and parameters collected during daily clinical routine (e.g., standard laboratory parameters depicting renal function and infection parameters, transfusion requirements, and substitution of coagulation factor concentrates) were extracted from automated patient data management systems (CareVue [Agilent technologies] and the IntelliSpace Critical Care and Anesthesia patient data management system [ICCA; Philips GmbH, Healthcare, Vienna, Austria; system started on April 23, 2013]).

### Laboratory Assessment

Blood samples were drawn from indwelling arterial or central venous catheters. Except for TGA, all coagulation assays were performed immediately.

#### Conventional Coagulation Assays (CCA) and Platelet Count

CCA were performed on citrated plasma (Vacuette® Greiner, Kremsmünster, Austria; trisodium citrate 3.8% 9:1 v/v). We assessed prothrombin time (PT; Owren, reference range: 70–125%), activated partial thromboplastin time (aPTT; reference range: 27–41 s), antithrombin (AT; reference range: 80–120%), fibrinogen (Clauss method; reference range: 200–400 mg dl^−1^), and antiXa levels (STA®-Liquid Anti-Xa, REF 00311 and REF 00322, reference range: <0.1 IU ml^−1^) via the STA R Max 2® coagulometer (Diagnostica Stago SAS, Asnières-sur-Seine, France). Platelet count (reference range: 150–350 G l^−1^) was determined from an EDTA tube (Vacuette® Greiner, Kremsmünster, Austria) via the Sysmex XE- 2100 cell counter (Sysmex, Kobe, Japan).

#### Thrombin Generation Assay

Blood samples were drawn into citrate-theophylline-adenine-dipyridamol (CTAD) test tubes (Vacuette® Greiner, Kremsmünster, Austria; 9:1 v/v) to minimize the effect of circulating microparticles, and immediately centrifuged at 4,500 *g* for 15 min. Platelet-poor-plasma (PPP) was then stored at −80°C for subsequent testing.

**Figure 1:**
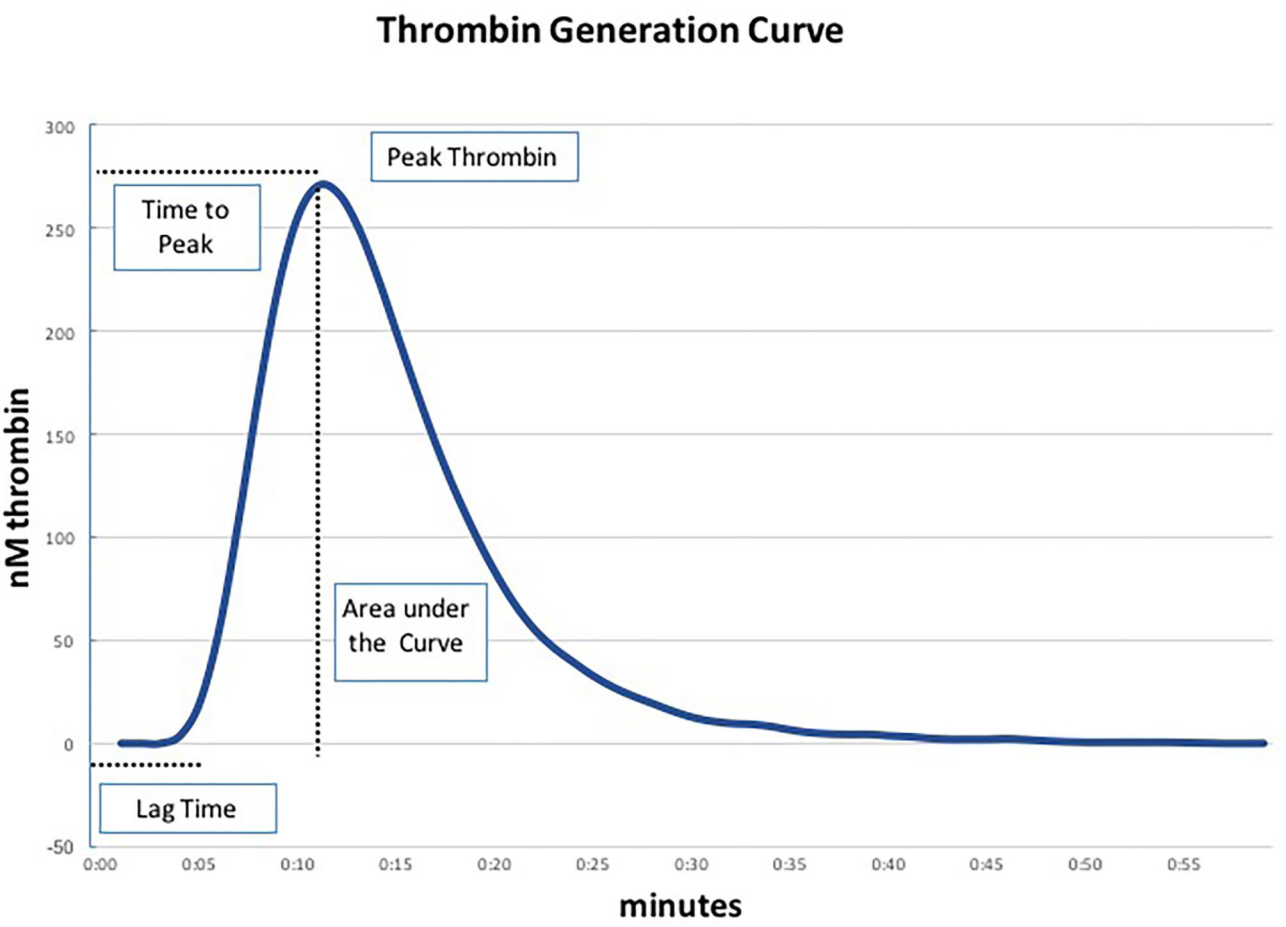
Parameters of the thrombin generation assay (TGA) (19).

We performed TGA using the fully automated Ceveron® alpha TGA analyzer (Technoclone, Vienna, Austria; Software Release V 2.1.2.2). For the conventional test, 15 μl reagent RC high (Ceveron® TGA RC high; Technoclone, Vienna, Austria), 35 μl CaCl_2_, and 20 μl reaction buffer are added to 40 μl PPP (thawed to 37°C) to initiate the coagulation process. Thrombin generated during the clotting process cleaves 40 μl of Z-Gly-Gly-Arg-AMC, a fluorogenic substrate (Ceveron® TGA substrate, Technoclone, Vienna, Austria). The concentration of thrombin is detected and plotted against time, resulting in a thrombin generation curve characterized by the following parameters: lag time (tLag, min), time to peak thrombin level (tPeak, min), and peak thrombin level (Peak, nM), after which the concentration of thrombin decreases (Figure 1). The velocity index (VI, nM min^−1^) is defined as Peak/(tPeak–tLag), the area under the curve depicts the endogenous thrombin potential (ETP, nM) (11). As PPP lacks endothelial cells containing thrombomodulin (TM), we performed a modified test in which recombinant human TM (Sekisui Diagnostics, LLC, Stamford, USA) is added at a concentration of 2 nmol L^−1^ to activate the protein C pathway and detect both pro- and anticoagulant determinants of hemostasis. In each blood sample ETP was determined using the conventional test assay (without thrombomodulin) and the TM-modified test assay in duplicate.

To better understand the pro- and anticoagulant forces in our patient cohort, we related ETP levels determined in the conventional assay (ETP_−*TM*_) to those obtained from the TM-modified assay (ETP_+TM_). ETP was then expressed as “ETP ratio” and calculated both for patients (ETP ratio patient) and standardized plasma of healthy donors (ETP ratio standardized plasma) (Technofrozen Control N, REF 5021100, Technoclone, Vienna, Austria). Standardized plasma of healthy donors was obtained from pooled plasma after plasmapheresis and fulfilled the following criteria: PT 75–150%, FVIII 75–150%, fibrinogen 200–450 mg dl^−1^. Higher ratios of ETP_+TM_ /ETP_−*TM*_ – mirroring a certain resistance to the anticoagulant activity of TM – were interpreted as increased procoagulant imbalance (12).

To enhance the sensitivity and reproducibility of TM-modified measurements, we followed the recommendation of Tripodi (12) and used the following formula to calculate a “normalized ETP ratio,” comparing ETP ratios of patients to those of standardized plasma:

$$\begin{array}{l} ETP-TM\;ratio\;Normalisation\\ =\frac{{ETP-PT}_{TM}}{{ETP-PT}_{-TM}}/\frac{{ETP-NM}_{TM}}{{ETP-NM}_{-TM}} \end{array}$$

ETP-PT_TM_, endogenous thrombin potential determined in the individual patient in the presence of thrombomodulin; ETP-PT_−*TM*_, endogenous thrombin potential determined in the individual patient in the absence of thrombomodulin (= conventional TGA); ETP-NM_TM_, endogenous thrombin potential determined from pooled normalized plasma samples in the presence of thrombomodulin; ETP-NM_−*TM*_, endogenous thrombin potential determined from pooled normalized plasma samples in the absence of thrombomodulin (= conventional TGA).

#### Multiple Electrode Aggregometry (Multiplate®)

Platelet function testing was conducted using tubes containing 25 μl ml^−1^ hirudin (Vacuette™ Greiner, Kremsmünster, Austria). MEA was started after blood samples had rested for 30 min, using the Multiplate® analyzer (Roche Diagnostics GmbH, Vienna, Austria). Details of the test procedure have previously been described (20). Commercially available test reagents (Roche Diagnostics GmbH) containing either arachidonic acid (ASPItest, reference range: 71–115 U), adenosine diphosphate (ADPtest, reference range: 57–113 U), collagen (COLtest, reference range: 72–125 U), or thrombin receptor activating peptide-6 (TRAPtest; reference range: 84–128 U) were added to saline-diluted whole blood to activate particular platelet receptors and initiate platelet aggregation.

### Diagnosis of VTE

Compression ultrasound of the lower extremities was performed for research purposes only, prior to inclusion in the study, on day C, and at discharge from the ICU. A lack of compressibility on B-mode ultrasound of either the common femoral vein or the venous system down to the popliteal vein was considered as proximal DVT. Pulmonary embolism (PE) was included if computed tomography pulmonary angiogram confirmed central/lobar PE. Owing to the lack of clinical relevance (21, 22), we did not account for distal/superficial DVT or subsegmental PE.

### Statistical Analysis

The central tendency and dispersion of continuous variables are described by median, and first and third quartiles, respectively. Gender differences in continuous demographic variables are assessed by Mann-Whitney-*U*-tests. Differences between measurements of continuous variables at baseline and day C were tested using Wilcoxon signed rank tests for paired samples. The same testing procedure applies to the comparison of TGA measurements in the presence and absence of thrombomodulin. All tests were two-sided, and *p*-values < 0.05 were considered statistically significant. All statistical analyses were performed with the statistical software R version 4.02 (R Core Team 2020) (23).

## Results

### Patient Characteristics

We prospectively enrolled 36 patients. Since therapeutic anticoagulation/platelet inhibition was started during CRRT in seven patients and TGA was not performed from CTAD tubes in all patients, 26 patients remained for analysis of data. Table 1 presents the demographic data, severity of critical illness, and risk of thrombosis which did not differ significantly between males and females. ICU data including CCA and standard laboratory parameters of renal function are presented at baseline and throughout day A–C in Table 2. We did not observe clinically relevant venous thromboembolism.

**Table 1:** Demographic data.

	**Male**	**Female**	***p*-value**
Sex (m/f)	13	13	na
Age (y)	66 (63;72)	65 (56;73)	0.94
BMI	25.0 (24.5;27.8)	28.4 (25.0;31.1)	0.24
SAPS III	60 (56;69)	71 (62;80)	0.13
Caprini Score	9 (7;10)	8 (6;9)	0.24

*Presented as median (1st quartile; 3rd quartile); p-values from hypothesis tests for gender differences; BMI, body mass index; SAPS III, Simplified acute physiology score 3; m, male; f, female; y, years; na, not applicable*.

**Table 2:** ICU data at baseline and during the first 72 h of CVVHD.

**Baseline**		**Day A**	**Day B**	**Day C**	*p*-value^*^
**Standard laboratory parameters referring to renal function and infection**					
BUN (mg dl^−1^)	65.5(35.2;99.5)	43.7(28.8;60.3)	26.3(22.8;38.0)	23.3(19.7;30.7)	<0.001
Creatinine (mg dl^−1^)	3.07(2.35;4.42)	2.03(1.77;2.69)	1.45(1.22;2.00)	1.32(1.04;2.15)	<0.001
CRP (mg dl^−1^)	18.9(8.0;25.3)	19.8(11.6;30.2)	19.0 (11.1;26.8)	16.0(10.2;24.6)	0.73
WBC (G l^−1^)	12.3(9.4; 18.9)	11.8(8.0;17.7)	11.7(7.3;17.2)	12.4(6.4;16.9)	0.44
**Coagulation assays and platelet count**					
PT (%)	60(48;85)	70(51;86)	73(56;91)	77(64;90)	0.12
aPTT (s)	39.3 (37;47.7)	42.0(38.6;46.3)	40.2(38.4;45.6)	38.6(35.4;42.8)	0.94
Fibrinogen (mg dl^−1^)	516(395;651)	562(391;687)	585(450;687)	584(490;653)	0.79
AT III (%)	63(46;95)	68(51;90)	71(58;92)	83(64;95)	0.05
AntiXa (IU ml^−1^)	<0.1 (<0.1;0.1)	<0.1(<0.1;0.16)	<0.1(<0.1;0.16)	<0.1(<0.1;0.14)	0.82
Platetet count (G l^−1^)	167(105;221)	168(97;201)	140(98;184)	141(114;188)	0.1
**Procoagulant drugs and transfusion requirements (*****n*****, number of patients receiving at least 1 application/unit)**					
TXA	0	0	0	0	na
Fibrinogen concentrate	1	1	0	0	na
PCC	1	0	0	0	na
Others	0	3	0	0	na
PRBC	6	6	2	1	na
Platelet concentrates	1	2	0	2	na
FFP	1	0	1	0	na
**Clinical outcomes**					
DVT and/or PE	0				na

*Data are presented as median (1st IQR;3rd IQR) or number of patients (n); BUN, blood urea nitrogen (reference range 6–23 mg dl^−1^); CRP, C-reactive protein (reference range <0.5 mg dl^−1^); WBC, white blood cells (reference range 4–10 G l^−1^); creatinine (reference range: 0.50–0.90 mg dl^−1^ for women and 0.70–1.20 mg dl-1 for men); TXA, tranexamic acid; PCC, prothrombin complex concentrate; PRBC, packed red blood cells; FFP, fresh frozen plasma; PE, pulmonary embolism; DVT, deep vein thrombosis; g, gram; d, day; ml, milliliter; s, seconds; mg, milligram; dl, deciliter; G, giga; l, liter*;

* *p-values from hypothesis tests for differences between results at baseline and day C*.

### Thrombin Generation Before and During CVVHD With Regional Citrate Anticoagulation

Table 3 presents the results of TGA as determined by the conventional and the TM-modified test assay.

**Table 3:** Parameters of thrombin generation (TGA) in patients at baseline and during CVVHD.

**tLag (min)**		**tPeak (min)**	**Peak (nM)**	**ETP (nM)**	**VI (nM min^**−1**^)**
**TGA**					
Baseline	3.6(3;4.9)	8.8(6.6;11.6)	184 (111;336)	2,105(1,155;2,757)	40(19;94)
Day A	3.4(2.8;4.3)	8.2(7.2;10.8)	178(86;323)	1,825(1,187;2,762)	38(13;67)
Day B	3.5(2.6;4.2)	7.8(6.7;10.7)	238(116;434)	2,146(1,313;3,528)	54(20;101)
Day C	3.6(2.7;4.2)	8.2(6.7;10.8)	254(170;382)	2,427(1,665;3,173)	55(26;101)
*p*-value^*^	0.04	0.13	0.31	0.42	0.42
**TM-modified TGA**					
Baseline	3.3(2.6;3.7)	6.8(5.8;8)	123(55;221)	972(533;1,689)	35(14;68)
Day A	3.1(2.6;3.6)	7.1(6.3;8.9)	117(42;197)	1,177(466;1,895)	31(8;59)
Day B	3.1(2.6;3.5)	6.8(5.8;7.9)	152(47;278)	1,250(446;2,308)	37(11;82)
Day C	3.1(2.7;3.5)	7.1(6.1;8.5)	160(56;287)	1,455(536;2,173)	45(13;81)
*p*-value^*^	0.4	0.5	0.33	0.65	0.3

*Data are presented as median (1st quartile, 3rd quartile); tLag, lag time; tPeak, time to peak; Peak, peak thrombin; ETP, endogenous thrombin potential; VI, velocity index; min, minutes; nM, nanomolar*;

* *p-values from hypothesis tests for differences between results at baseline and day C*.

**Figure 2:**
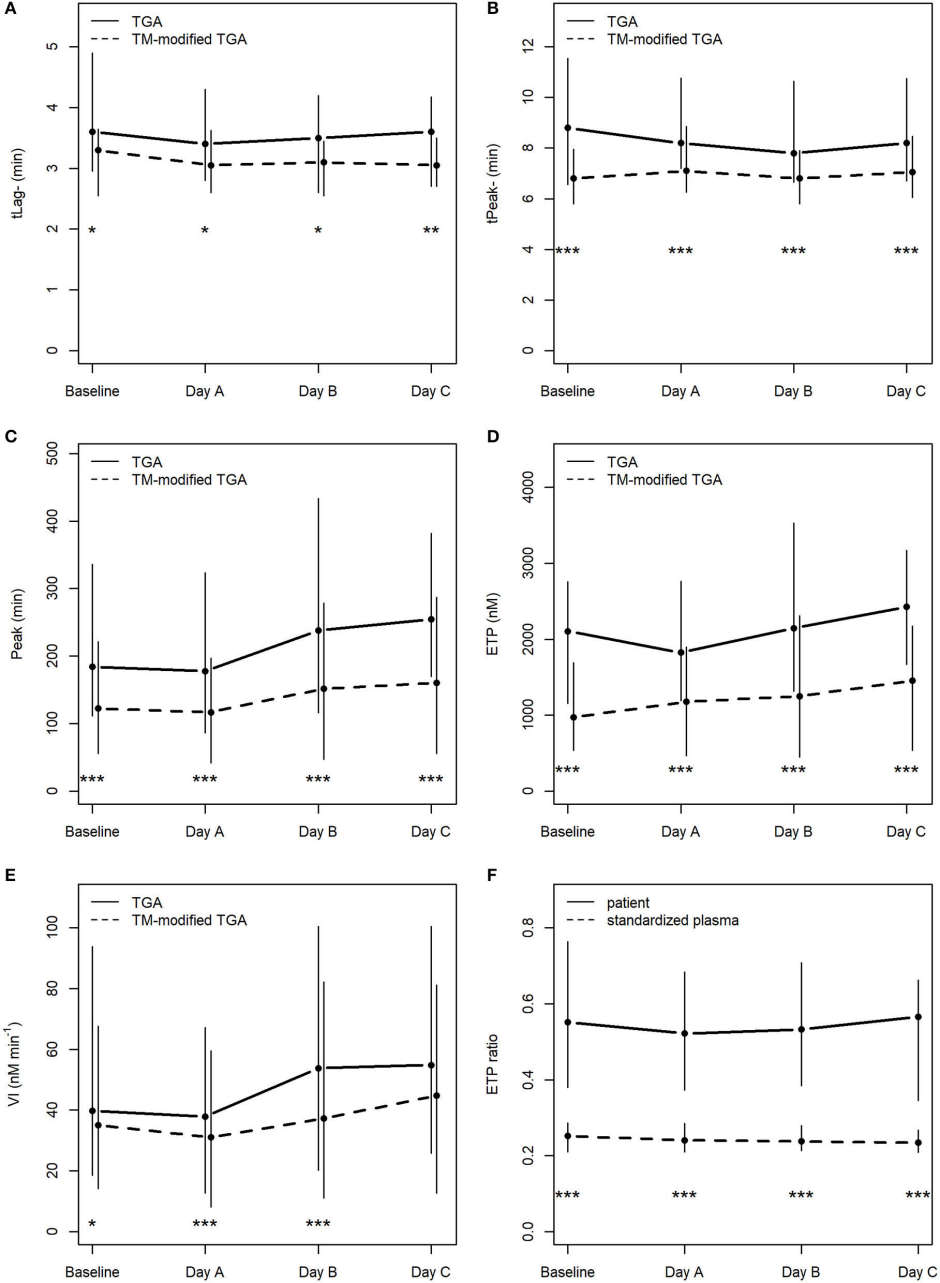
Course of TGA parameters in the conventional TGA and the TM-modified assay **(A–E)**, **(F)** depicts ETP ratios (ETP_+TM_ : ETP_−*TM*_) in patients and in standardized plasma of healthy donors. *p < 0.05; **p < 0.01; ***p < 0.001; tLag, lag time; tPeak, time to peak; Peak, peak thrombin; ETP, endogenous thrombin potential; VI, velocity index; min, minutes; nM, nanomolar.

As expected, TGA parameters differed significantly when thrombomodulin was added to the test procedure (Figures 2A–E).

There was no statistically significant change in any of the TGA parameters during the course of CVVHD (baseline to day C) (Table 3). However, peak thrombin and ETP levels increased over time in the conventional as well as the TM-modified assay.

Calculated median ETP ratios for patients remained stable over the study period, with 0.55 [0.38; 0.76] at baseline, 0.52 [0.37; 0.68] at day A, 0.53 [0.38; 0.71] at day B, and 0.57 [0.34; 0.66] at day C. Results at baseline did not differ significantly from those obtained at day C (*p* = 0.59).

ETP ratios were significantly higher (*p* < 0.001) in patients when compared to standardized plasma of healthy donors (baseline: 0.25 [0.21; 0.29]; day A 0.24 [0.21; 0.29]; day B 0.24 [0.21; 0.28]; day C 0.23 [0.21; 0.27]). Figure 2F illustrates the course of ETP ratios in patients and standardized plasma.

With respect to ETP ratios of patients compared to those generated from standardized plasma (“normalized ETP ratio”), the following results can be reported: 2.17 [1.42; 3.02] at baseline, 2.04 [1.65; 2.86] at day A, 2.24 [1.76; 2.89] at day B, and 2.66 [1.58; 3.08] at day C. There was no significant change from baseline to day C (*p* = 0.27).

### Platelet Function Determined via MEA Before and During CVVHD With Regional Citrate Anticoagulation

**Figure 3:**
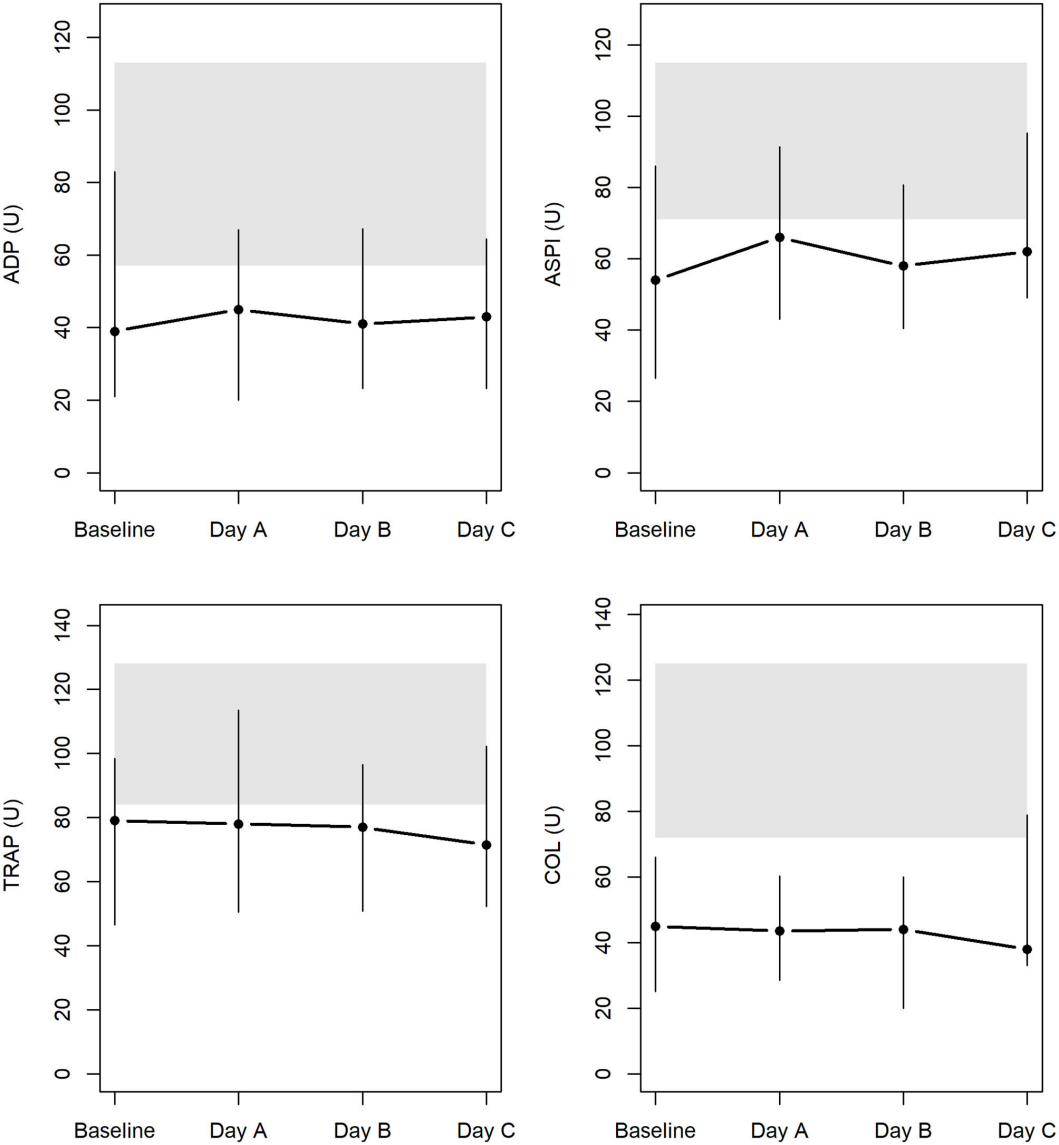
Platelet function at baseline and during CVVHD with regional citrate anticoagulation (gray bars highlight reference ranges provided by the manufacturer at time of measurements).

Results of the AUC revealed decreased platelet function, with values below defined reference ranges at baseline and the following 3 days during CVVHD (Figure 3). Although we observed a significant decline in creatinine and blood urea nitrogen (*p* < 0.001) during CVVHD, proving efficacy of the extracorporeal therapy, platelet function did not change significantly. Table 4 presents detailed test results during the observational period.

**Table 4:** Results of Multiplate during CVVHD: no significant effect on platelet function despite successfully treated uremia.

**Multiplate**	**Baseline**	**Day A**	**Day B**	**Day C**	*p*-value^*^
ADP *(U)*	39(21;83)	45(20;67)	41(23;67)	43(23;65)	0.84
ASPI *(U)*	54(27;86)	66(43;92)	58(41;81)	62(49;95)	0.17
COL *(U)*	45(25;66)	44(29;60)	44 (20;60)	38(33;79)	0.57
TRAP *(U)*	79(47;99)	78(51;114)	77(51;97)	72(52;102)	0.3
BUN *mg dl^−1^*	74.0(37.1;99.6)	42.0(28.1;61.1)	26.2 (22.1;37.0)	22.8(18.6;29.8)	<0.001

*Data are presented as median (1st quartile, 3rd quartile); BUN, blood urea nitrogen (reference range 6–23 mg dl^−1^); U, unit (= area under the curve)*;

* *p-values from hypothesis tests for differences between results at baseline and day C*.

## Discussion

In this study we did not observe significant changes in thrombin generation after the start or during the course of CVVHD using regional citrate anticoagulation. Test results for the conventional TGA differed significantly from those for the TM-modified assay. ETP ratios in patients were significantly higher compared to the standardized plasma of healthy donors confirming the assumed procoagulant alterations in ICU patients. Taken together, this could be interpreted as the CVVHD anticoagulated with citrate does not further aggravate the *per se* procoagulatory shift in the ICU patient. Moreover, throughout the whole study period, platelet dysfunction did not improve despite the successful treatment of uremia.

TGA has recently attracted attention in hemostatic research as it allows hypo- and hypercoagulable alterations to be detected (11, 24) – both of which are present in critically ill patients. To date, data on TGA in critically ill patients have been reported in the context of liver failure, trauma, sepsis, severe burn injury, and extracorporeal membrane oxygenation (4, 19, 25–27). Moreover, TGA is increasingly used to investigate the efficacy of anticoagulant therapy beyond CCA, also during critical illness (28–31).

To the best of our knowledge, we are the first to describe TGA in patients with severe kidney injury requiring extracorporeal organ support with regional citrate anticoagulation.

Petros and colleagues performed conventional and TM-modified TGA in patients with severe sepsis (26). As in our study, tests were performed at baseline (within 24 h of the diagnosis of severe sepsis) and with a follow-up (by day 8). The parameters of conventional TGA differed significantly at baseline compared to healthy controls, except for ETP levels. Lag time, time to peak, and peak thrombin were lower in non-survivors in both the conventional and the TM-modified assay. Unfortunately, the results are not comparable to our study owing to the different TM concentration used (5 pM in the final solution).

Gouya and colleagues performed TGA to describe the bioactivity of enoxaparin beyond conventional antiXa measurement in 16 critically ill patients (31). Peak thrombin levels measured with RC high reagent were much higher at baseline (344 nM, IQR 150; 657) and 3 h after administration of LMWH (269 nM, IQR 34; 482) than in our study. In contrast, the authors found ETP levels (median at baseline ~750 nM; median 3 h after administration ~630 nM) far below those we found in our patients. It should be noted that normal renal function was a precondition for participation in this study.

Also of note, ETP ratios comparing ETP measured in the presence and absence of TM have not yet been reported in either of these patient cohorts.

ETP measurement is affected by temperature and concentration of tissue factor and phospholipids (12). For that reason, in 2017 Tripodi appealed for the careful standardization of ETP by calculating ETP ratios (12). The ETP-TM ratio represents the resistance to the anticoagulant activity of TM (12). The higher the ratio, the greater the procoagulant imbalance (12). ETP-TM ratios remained stable during the observation period of our study, but were significantly higher in patients compared to the standardized plasma of healthy volunteers, indicating a greater procoagulant potential. Tripodi also postulated the interpretation of the ETP-TM ratio in relation to ETP levels of standardized plasma of a healthy control group to enhance sensitivity and reproducibility of TM-modified measurements (12). When the ETP ratio was compared to standardized normal plasma samples in our cohort, we observed an increase in ratio from baseline until day C. The calculation of ETP ratios has not yet become standard; ETP ratios have been reported in cirrhotic patients, patients with polycythemia vera, essential thrombocytopenia, and idiopathic myelofibrosis, but not in critically ill patients (12).

In addition to TGA, we performed MEA at the uremic state and tracked platelet function throughout CVVHD. The AUC revealed an impaired platelet function affecting platelet activation via arachidonic acid, adenosine diphosphate, collagen, and TRAP-6-related pathways at baseline. Test results remained below defined reference ranges despite efficient elimination of urinary excreted substances.

Underlying pathomechanisms of uremia-related platelet dysfunction (e.g., diminished release of thromboxane A_2_, altered composition of surface receptors resulting in decreased interaction with the endothelium, other platelets, and fibrinogen) are complex and have been described previously (9, 15, 32). Data on platelet function in uremic patients are also available for MEA. Gäckler and colleagues performed MEA in non-critically ill patients undergoing intermittent hemodialysis due to chronic end-stage renal disease (33). Reduced AUC levels were found only in some patients: 25% of patients showed reduced AUC in the ADP test, 45% in the ASPI test, and 10% in the TRAP test (33). Notably, the test results are not comparable to data we found in our study, as the type of hemodialysis and anticoagulation differ. Wand and colleagues investigated MEA in a partly comparable setting to ours: Multiplate® performed in patients with acute kidney injury before start of CRRT and 6, 12, 24, and 48 h after initiation of CRRT (34). Similar to our results, the authors did not find a significant change in platelet function over time during CRRT, except for one significant drop in AUC in the ASPI test 6 h after initiation of CRRT (34). In contrast to our results, the median AUC of the TRAP and ASPI assay remained within the defined reference ranges for the test. However, here too, the ADP test revealed persistent impaired platelet function (34). As the reported reference ranges differ from ours, we can assume a different composition of reagents was used, explaining why the absolute values are not comparable (34).

It is worth noting that median platelet count in our patient cohort remained above 100 G L^−1^ which, to date, is the recommended lower limit for achieving reliable test results in point-of-care platelet tests such as Multiplate®^1^.

The following limitations should be considered: firstly, TGA still lacks defined reference values; and test reagents vary between studies. To achieve some degree of standardization, we followed the recommendation to compare the test results from patients to those from the standardized plasma of healthy donors. Secondly, the results of MEA did not allow us to draw conclusions with respect to the potential underlying pathomechanisms of platelet dysfunction in our patient cohort. We performed MEA to rule out the potential impact of platelets on prohemostatic alterations and did not intend to fully elucidate platelet function in this defined context. Thirdly, for a variety of reasons, the study inclusion took several years. However, both, the material and standard procedures for CVVHD, as well as the anticoagulant regimes, were identical throughout the entire study period.

In summary, in-depth analysis using standard and modified TGA, as well as the calculation of ETP ratios, revealed no further aggravation of the procoagulatory shift in the critically ill patient by/during CVVHD using regional citrate anticoagulation. MEA ruled out the potential impact of platelets.

## Data Availability Statement

The original contributions presented in the study are included in the article/Supplementary Material, further inquiries can be directed to the corresponding author.

## Ethics Statement

The studies involving human participants were reviewed and approved by Ethics Committee of the Medical University of Vienna (4 July 2012, No. 1416/2012). The patients/participants provided their written informed consent to participate in this study.

## Author Contributions

MW and ES contributed to the study conception and design. MW, LI, CL, and SK performed material preparation and data collection. AB performed statistical analysis. MW and ES wrote the first draft of the manuscript and all authors commented on previous versions of the manuscript. All authors read and approved the final manuscript.

## Conflict of Interest

MW received travel reimbursement and speaker's fees from Boehringer Ingelheim and CSL Behring for lecturing activities. The remaining authors declare that the research was conducted in the absence of any commercial or financial relationships that could be construed as a potential conflict of interest.

## Footnotes

^1^https://diapharma.com/wp-content/uploads/2016/07/Multiplate_Package_Inserts_R5_ML-02-00011-.pdf.
