# Supplementary figures and images for: Thrombin Generation and Platelet Function in ICU Patients Undergoing CVVHD Using Regional Citrate Anticoagulation
Source: Front Med (Lausanne). 2021 Jun 14;8:680540. doi: 10.3389/fmed.2021.680540 (PMC8238086; doi:10.3389/fmed.2021.680540)

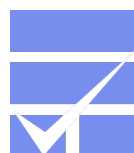

# CONSORT

TRANSPARENT REPORTING of TRIALS

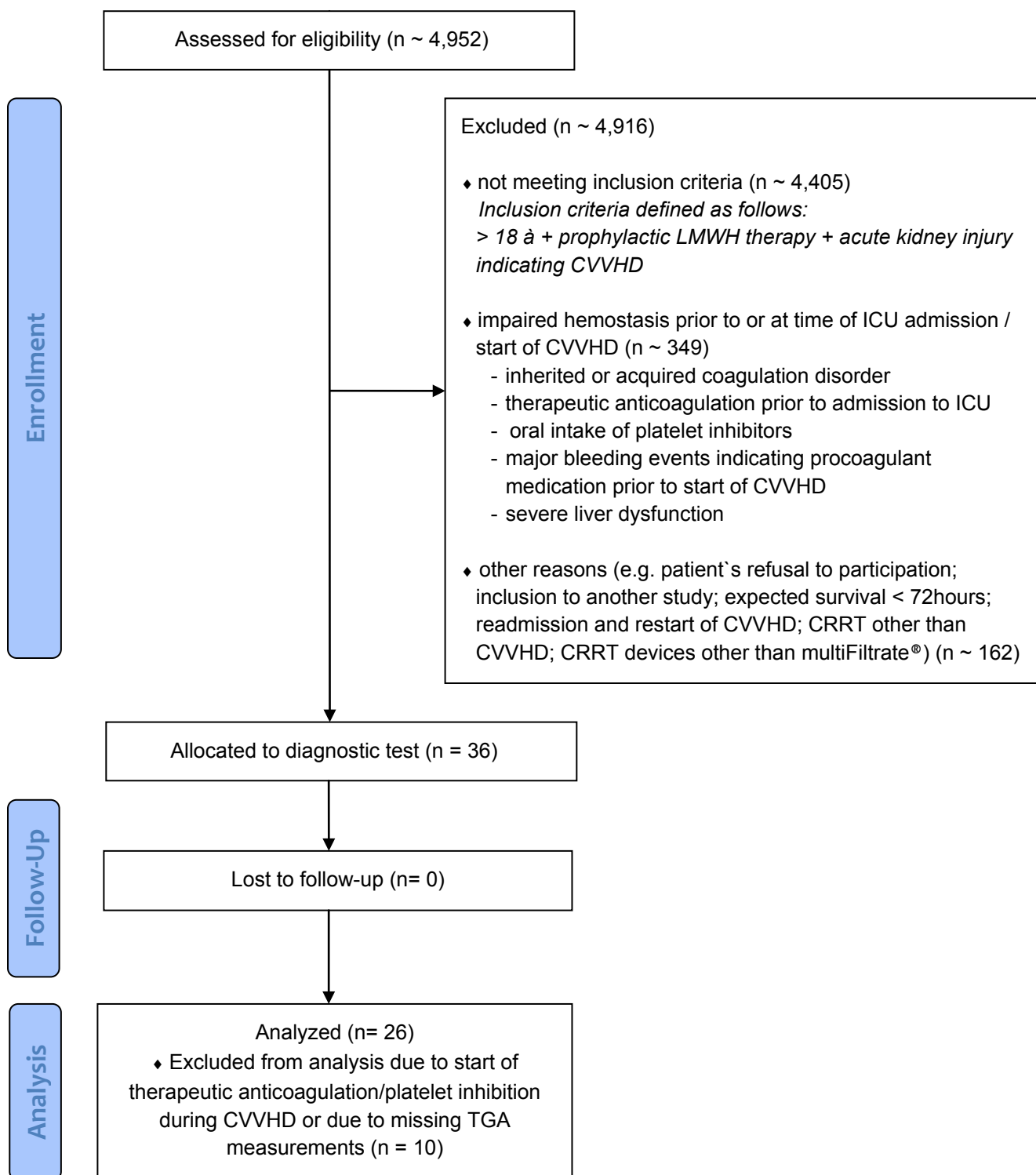

Supplement: Supplementary file 1 [file Data_Sheet_1.pdf]
